# Transcriptome map of plant mitochondria reveals islands of unexpected transcribed regions

**DOI:** 10.1186/1471-2164-12-279

**Published:** 2011-06-01

**Authors:** Sota Fujii, Takushi Toda, Shunsuke Kikuchi, Ryutaro Suzuki, Koji Yokoyama, Hiroko Tsuchida, Kentaro Yano, Kinya Toriyama

**Affiliations:** 1 Laboratory of Environmental Biotechnology, Graduate School of Agricultural Science, Tohoku University, Sendai 981-8555, Japan; 2 Graduate School of Science, Kyoto University, Oiwakecho, Kitashirakawa, Sakyo-ku, Kyoto 606-8502, Japan; 3 School of Agriculture, Meiji University, Kawasaki 214-8571, Japan

## Abstract

**Background:**

Plant mitochondria contain a relatively large amount of genetic information, suggesting that their functional regulation may not be as straightforward as that of metazoans. We used a genomic tiling array to draw a transcriptomic atlas of *Oryza sativa japonica *(rice) mitochondria, which was predicted to be approximately 490-kb long.

**Results:**

Whereas statistical analysis verified the transcription of all previously known functional genes such as the ones related to oxidative phosphorylation, a similar extent of RNA expression was frequently observed in the inter-genic regions where none of the previously annotated genes are located. The newly identified open reading frames (ORFs) predicted in these transcribed inter-genic regions were generally not conserved among flowering plant species, suggesting that these ORFs did not play a role in mitochondrial principal functions. We also identified two partial fragments of retrotransposon sequences as being transcribed in rice mitochondria.

**Conclusion:**

The present study indicated the previously unexpected complexity of plant mitochondrial RNA metabolism. Our transcriptomic data (*Oryza sativa *Mitochondrial rna Expression Server: OsMES) is publicly accessible at [http://bioinf.mind.meiji.ac.jp/cgi-bin/gbrowse/OsMes/#search].

## Background

The obvious expansion of its genomic size indicates that higher plant mitochondria experienced a dramatic evolution. The common size of mitochondria genetic information is limited to approximately 16 kb in metazoans [1], whereas in higher plants the sequence length can be 200-2400 kb (Additional file 1) [2]. The principal role of mitochondria (i.e. oxidative phosphorylation) is undoubtedly shared between metazoans and higher plants. The presence of three genes encoding subunits of ATP synthase (*atp6 *and *atp8*), three genes encoding subunits of cytochrome oxidase (*cox1-cox3*), *cytochrome b *(*cob*), and seven genes for NADH dehydrogenase (*nad1-nad4*, *nad4L*, *nad5 *and *nad6*) is indeed conserved in mitochondria of both kingdoms. What is extra in plant mitochondria compared to that of metazoans are only a few more respiratory-related genes (including *atp1*, *atp9*, *nad7 *and *nad9*) and dozens of genes encoding ribosomal subunits (*rps *or *rpl*). Thus, usually a higher plant mitochondrion encodes about 40 genes with known functions, whereas in most cases there are 13 tightly conserved genes encoded by metazoan mitochondria. Thus, a greater number of mitochondrial genes would explain only a small proportion of the genome size increase of plant mitochondria.

A partial answer to the mysterious expansion of mitochondrial genome size was given by recent plant mitochondrial genome sequencing studies. According to NCBI (http://www.ncbi.nlm.nih.gov), mitochondrial genome sequencing of 14 Magnolyophyta species is currently complete. These include dicot species *Arabidopsis thaliana *[3], *Beta vulgaris *[4,5], *Brassica napus *[6] and *Nicotiana tabacum *[7]; and monocots *Oryza sativa *[8-10], *Zea mays *[11,12] and *Triticum aestivum *[13]. These studies not only reported the great variability in size and gene content of mitochondria among species, but also even within species mitochondrial genomic structure can vary significantly. For example, different *Z. mays *lineages can carry mitochondrial genomic information of range 536-740 kb [11,12]. Much of the size differences of these genomes are due to different numbers of large genomic duplication events, and apparent genomic recombination events between lineages. The comparison of a *B. vulgaris *cytoplasmic male sterile (CMS) strain with the non-CMS strain showed a complex rearrangement of sequence blocks [4,5]. We recently sequenced two CMS strains of rice (*O. sativa *and *O. rufipogon*), and at least 12 genomic recombination events were necessary to explain the origin of the mitochondrial genome compared to the reference genome [8].

To understand the nature of large inter-genic region, we used a 60-mer probe-tiling array to visualize the expression pattern of the entire rice mitochondrial genome. In calli, 48.5% of the regions could be regarded as being transcribed. By setting the transcriptional borders by defining transcriptional units, we showed that 36.9% of open reading frames (ORFs) present in inter-genic regions were being transcribed without association with known mitochondrial housekeeping genes. We also identified two different partial fragments of transposable elements (TEs) that were being transcribed, suggesting unexpected complexity of transcriptional regulation in plant mitochondria.

## Results

### Experimental design

The mitochondrial genome size of Nipponbare, the rice cultivar commonly used in molecular biology, is estimated at 490 520 bp [9]. Tiling probes of 60 mer were designed for 374 866-bp non-redundant sequences after discarding large duplicated regions of > 10 000 bp. The probes overlapped each other by 58 nucleotides, meaning that tiling probes with 2-bp intervals were designed for the 374 866-bp region. Mitochondrial RNA was prepared from calli or etiolated-seedlings and hybridized against the tiling probes after biotin labeling upon reverse transcription by random primers. For further information on the processing of tiling array output, such as expression value normalization, see Methods.

### Identification of transcribed region

**Figure 1:**
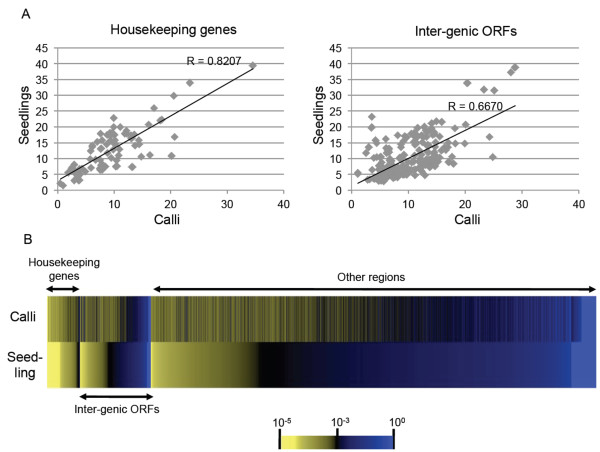
**Rice mitochondrial transcriptome**. **(A) **Tissue-specific plot of mean RNA expression level. Each dot represents each genic region. Housekeeping genes are known mitochondrial genes such as subunits of ATP synthetase, cytochrome oxidase or ribosomal proteins. Inter-genic ORFs are ORFs located between known housekeeping genes. **(B) **Confidence of expression of each type of regions. Each box represents the expressional confidence level (*p-*values by Student's *t*-test) as described in Methods. For simplicity of the figure, 8000 probes at even intervals were selected from the entire genome for display. Selected probes were categorized into three types, depending on which region of the genome they were located on (housekeeping genes, inter-genic ORF region or region without any ORFs predicted). Probes were arranged in descending order of expressional confidence in seedlings, for each category of genomic regions (housekeeping genes, inter-genic ORFs and other regions).

To restrict the transcribed region, we used the relative transcription levels of *orf490 *and *orf181 *as background (untranscribed) controls. Transcripts of these two ORFs were not detected in our preliminary transcription analysis by northern blotting (Additional file 2). These genes are partly TE fragments, making them highly unlikely to have any function in mitochondria, and therefore we concluded that these genes were not transcribed. A sliding window was set as a cluster of 60 consecutive probes, and normalized expression values of each sliding window were compared against 1124 probes spanning the regions of *orf490 *and *orf181*. If the mean value of each window significantly exceeded the mean value of the 1124 probes (Student's *t*-test, *p *< 0.001), the window was considered to represent an expressed region. As a result, we estimated that 48.5% of the mitochondrial genome was being transcribed in rice calli, compared to only 32.0% in etiolated seedlings. The discrepancy between these values is mostly explained by being a region without annotations of previously known mitochondrial housekeeping genes. For simplicity we designated the region without previous annotations by known mitochondrial genes the 'inter-genic' regions. Positive correlations of RNA expression between the two tissues (i.e. calli and seedlings) were higher in the previously annotated housekeeping genes (*r *= 0.82, *p *< 10^-21^; d.f. = 84) than in the ORFs predicted by six-frame translation in inter-genic regions (*r *= 0.67, *p *< 10^-25^; d.f. = 301) (Figure 1A). This indicates that RNA expression regulation of known housekeeping gene models was well conserved between the two tissues, while expression of the inter-genic region was less conserved.

All regions carrying known mitochondrial genes were regarded as being transcribed under the above statistics we used (Figure 1B). Almost all probes within the known genic regions gave *p *< 0.001, thus, the criterion we used to choose the expressed region was correct at least in this sense.

### Transcriptional units in inter-genic regions

**Figure 2:**
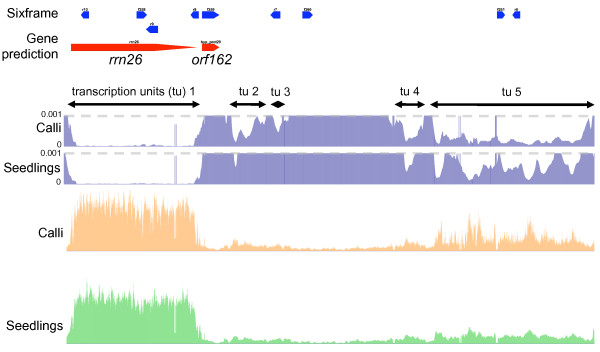
**Example of TU and transcript mapping**. Upper two rows indicate the confidence of RNA expression (*p-*values) as described in Methods. Orange bars indicate the normalized RNA expression level in calli, and green bars indicate that of seedling tissues.

To comprehensively reveal the RNA expressional regulation of inter-genic regions, it was necessary to make a clear distinction between transcripts that were associated with known housekeeping genes (e.g. untranslated region), and the transcripts that were not associated with these gene features. Therefore, we designated each continuously expressed region (≥ 20 consecutive probes) as a 'transcriptional unit' (TU; see Figure 2). This region contained a highly expressed 26 S ribosomal RNA gene (*rrn26*), and there was a high chance of RNA expression in this region (significantly higher than background control, *p *< 0.001), and of high scores for relative RNA expression level. These consecutive low *p-*value regions with a 'valley'-like transcriptional landscape were termed as single TUs in this study for brevity. Although there were relatively lower chances of RNA expression, this region contained four more TUs (tu2, tu3, tu4 and tu5; Figure 2) other than the *rrn26 *TU. Further details of TUs are described in the Materials and methods. As a result of TU searches using the *p *< 0.001 criterion, we detected 653 and 297 TUs in calli and seedlings, respectively. All TUs detected in seedlings were also identified in calli, suggesting that RNA regulation of calli was far more complex, or otherwise leaky, compared to that of seedlings.

**Figure 3:**
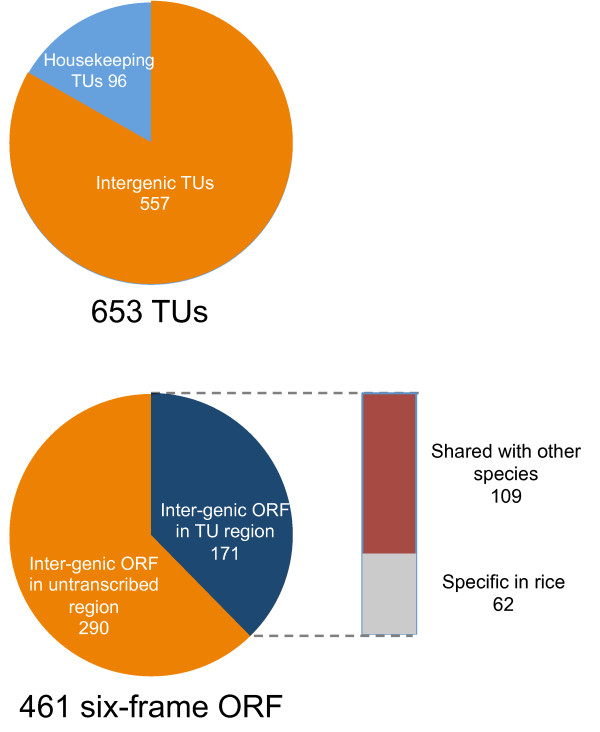
**Summary of TUs and inter-genic ORFs**.

To determine the meanings of TUs detected in inter-genic regions, we used six-frame translation to predict ORFs present within these TUs. Six-frame translation analysis predicted 505 ORFs in the rice mitochondrial genome and of these, 461 were not annotated as mitochondrial genes (Additional file 3). We designated the ORFs in inter-genic regions as inter-genic ORFs (iORFs). These 461 iORFs were numbered iORF_1 to iORF_505, skipping the numbers of frames corresponding to known genes (e.g. *atp1*) (Additional file 3). It should be noted that 25 ORFs were previously designated [9], but we will use our IDs throughout for brevity. Among these 461 iORFs, 171 were within one of the TUs that were not associated with housekeeping genes. Thus, of 557 TUs located in the inter-genic regions, 171 carried at least one protein frame longer than 70 amino acids (Figure 3).

**Figure 4:**
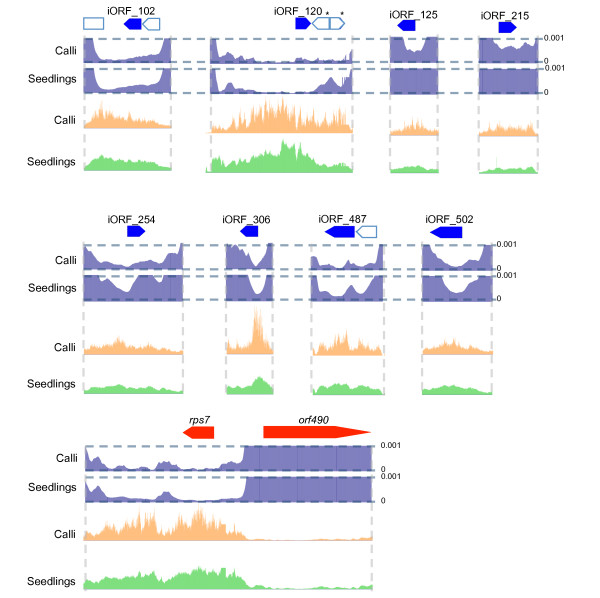
**Normalized RNA expression values in conserved inter-genic ORFs**. Colored boxes represent frames conserved among species, whereas uncolored boxes were not. RNA expression patterns of *rps7 *(expressed) and *orf490 *(unexpressed) are displayed for comparison.

**Figure 5:**
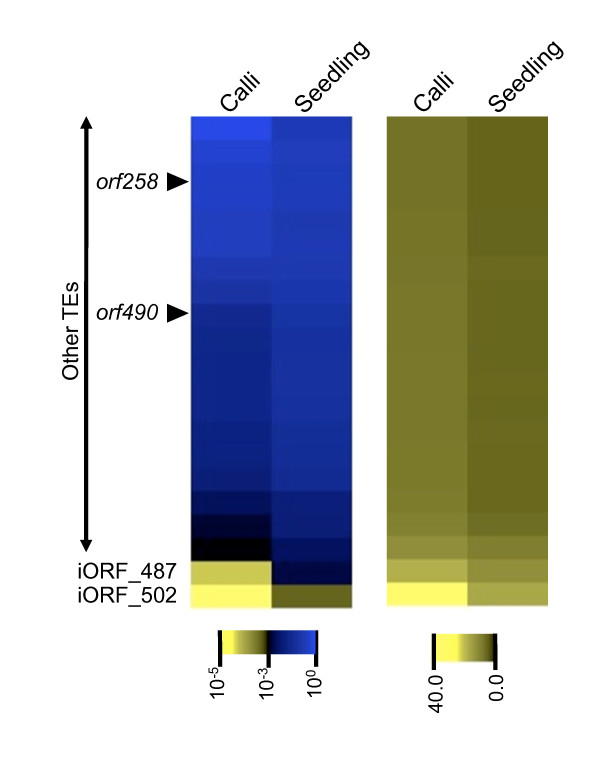
Confidence of expression (left, in *p-*value) and normalized expression levels (right) of putative TEs in mitochondria.

If the protein functions of these newly identified iORFs are important to mitochondrial function, we hypothesized that the extent of conservation of these ORFs among the species would be positively linked with their transcription. The idea is supported by the fact that conserved genes are generally highly expressed, whereas species-specific genes are expressed at low levels in specific tissue types [14]. Thus, to further understand the nature of these iORFs, we conducted a comprehensive database analysis. Using a bi-directional BLAST search, we sought for orthologous ORFs from the exact same six-frame translation protein libraries of mitochondrial genomes of 13 other land plant species. Although orthologous protein frames were found from these species, there was no statistically significant correlation between frame conservation and transcript expression. However, there were eight ORFs (iORF_102, iORF_120, iORF_125, iORF_215, iORF_254, iORF_306, iORF_487 and iORF_502; Figure 4) conserved among several species, especially among monocotyledons, and were transcribed in single TUs without associating other known gene features. An example of such ORFs was iORF_120, and the presence of this frame was shared among four other monocotyledon species: *Sorghum bicolor*, *Triticum aestivum*, *Tripsacum dactyloides *and *Z. mays*. As frames adjacent to iORF_120 (at the right side of iORF_120 with asterisks, Figure 4) were not present in these species, it was unlikely that iORF_120 was conserved among species as a side effect by synteny of large genomic fragments. Thus, we presume that iORF_120 may be a gene at least in monocotyledons and could possess a role in mitochondria. We confirmed the expression of these eight iORFs by RT-PCR analysis (Additional file 4). This proves that signal detection of these regions was not the result of cross-hybridization. Of the eight ORFs just mentioned, iORF_487 and iORF_502 shared similarities with TEs. According to Notsu et al. [9], 19 TEs (16 retrotransposons and three DNA transposons) are present in the rice mitochondrial genome. iORF_487 had significant similarity with a part of *A. thaliana *retroelement polymerase polyprotein (Additional file 5), and was previously identified [9]. iORF_502 had a significant similarity with part of another long terminal repeat (LTR) retrotransposon in bamboo (*Phyllostachys edulis*) (Additional file 5), and this frame was newly identified in the present study. These two fragments of TEs, iORF_487 and iORF502, were the only ones being transcribed among 20 partial TEs identified in total (Figure 5). Although these fragments were clearly transcribed, it was unlikely that these fragments were actively transposable, given that they carry only partial sequences of a retrotransposon. Non-autonomous transposing activity was equally unlikely, as LTRs were not present in these iORFs.

## Discussion

In this study we conducted a rice mitochondrial genomic-tiling array to reveal the hidden factors that expanded plant mitochondrial genomic information size. Depending on the tissue type, at most 48.5% of the mitochondrial genome was being transcribed. Transcription frequencies of iORFs can be dependent on tissue types (Figure 1). Several iORFs could be considered as transcribed not by coincidence, suggesting the hidden role of mitochondria. There are currently no further hints for functions of these iORF features conserved among species. This is because no similar protein motif was identified from non-redundant protein database searches, and it is not easy to speculate their protein function even if they were translated. Alternatively, there could be a possibility that these transcripts do not encode a protein. They could be transcribed as non-coding RNA (ncRNA) genes. This is supported by the fact that there are a few transcripts that do not carry ORF longer than 70 amino acids (Additional file 4). For example, ncRNA4 does not carry a reading frame longer than 42 codons. It is more convincing to consider that these transcripts are ncRNAs rather than to consider that they encode a protein. To date, no ncRNA-mediated gene regulation mechanism has been described in plant mitochondria. It is possible that these inter-genic transcribed regions serving the ncRNA are required for regulation of other genic regions.

One of the most important findings in the present study was the detection of transcription of two partial TEs. TE fragments have been found in all plant mitochondrial genomes sequenced so far [6,7,9,12,13]; however, most of them were not considered functional, as they were present only in fragments. One possibility is the detection of contaminated nuclear RNA. However, nucleotide identities of these two TEs (iORF_487 and iORF_502) with their respective nuclear sister copies were 59.3 and 63.2%, low enough to be washed off during the post-hybridization processing. Secondly, transcript detection of TE fragments from nuclear RNA contamination seems odd, when considering that there were 20 TE-like sequences present in mitochondria and that transcripts for 18 were not detected, even though they too possess decent numbers of nuclear sister copies. We have also confirmed the RNA expression of iORF_487 and iORF_502 by RT-PCR analysis (Additional file 4). Lastly, as unrestricted transposition of TEs may cause deleterious genomic mutations, the majority of nuclear TEs would be suppressed by DNA methylation [15]. Indeed, best sister copies of each mitochondrial TE fragment were not transcribed, according to the nuclear tiling array profile in the TIGR rice genome browser [16]. Therefore we consider that false-detection of a nuclear copy was unlikely in this case.

Although these TE fragments (iORF_487 and iORF_502) are unlikely to possess transposing activities given the incomplete fragments of TE protein sequences they carry (Additional file 5), it is likely that they are the remnants of past TE integration into the mitochondrial genome. Recent study of RNA editing site loss in *Silene *spp. has shown evidence of integration of reverse-transcribed mRNA into mitochondrial genome [17]. It is possible that similar re-integration events can occur for other mitochondrial RNA. In the present study we showed that the mitochondrial RNA pool is a mixture of various transcripts from unexpected places within the genome. Re-integration of such transcripts might have contributed to complexity of flowering plant mitochondria compared to other species. A good example is the chimeric structure of most CMS-related genes, which are often present as the fused chimeric protein of partial respiratory chain subunit and an unknown sequence [18]. The peptide region of an unknown sequence can originate from inter-genic transcripts; these sequences may be quickly lost during evolution and missing in extant species, while they can be retained as a part of CMS-related gene. This might be one explanation for why sequences of unknown origin are often found within CMS-related genes.

Approximately 50% of a typical plant mitochondrial genome does not show any similarities with other plant mitochondrial genomes [12], suggesting the involvement of frequent genomic recombination or horizontal transfer events in enlargement of the plant mitochondrial genome. It is also possible that as mitochondria alter their genomic arrangement quite frequently [5,8,11], nuclear-encoded RNA polymerases are not yet adapted to optimize the transcriptional efficiency of mitochondria. This could be the reason why so many inter-genic regions that are seemingly unrelated to mitochondrial functions are being transcribed by residual functions of un-optimized RNA polymerases.

## Conclusion

We conclude that the plant mitochondrion is enriched with unknown transcripts that are mapped on inter-genic regions. This is obviously related to the large genomic information size of plant mitochondria, and revealing the functions of these transcripts should improve our understanding of the expansion of the plant mitochondrial genome. Finally, the results presented here are publicly accessible as *Oryza sativa *Mitochondrial rna Expression Server (OsMES: http://bioinf.mind.meiji.ac.jp/cgi-bin/gbrowse/OsMes/#search).

## Methods

### Mitochondrial DNA extraction

Calli and seedling mitochondria were purified on sucrose gradients with an ultracentrifuge, as described by Tanaka et al. [19]. Three-week-old calli inducted from seeds were transferred to fresh medium, and mitochondria were isolated after one week of incubation. Seedlings were grown in complete darkness for two weeks before mitochondrial preparation. Total mitochondrial RNA was extracted by standard phenol/chloroform RNA isolation method.

### Construction of tiling array

Sixty-mer probes were designed for 374 866-bp non-redundant rice mitochondrial genomic sequences, with 2-bp intervals. Thus, a total of 374 866 probes were designed from sense and antisense directions. Probe synthesis and array construction was done in GeneFrontier (Tokyo, Japan). Cy3-labeled double-strand cDNA products of total mitochondrial RNA were obtained using a random primer. All of the hybridization and signal detection experiments were done in GeneFrontier. Custom perl scripts were written to re-map the probes and hybridization signals onto the 490 520-bp rice mitochondrial genome (BA000029). The tiling array data is deposited to Center for Information Biology Gene Expression database (CIBEX; http://cibex.nig.ac.jp) under accession no. CBX156.

### Statistical analysis of RNA expression

Signal intensities of sense and antisense probes were treated as experimental replicates. Normalization of each tissue was done by quantile-scaling using limma library in R [20]. Expression values were then converted into Z-scores. Expression levels of *orf490 *and *orf181 *were used as the background control, e.g. the reference for an untranscribed region. The 1124 probes spanning these regions were compared against every sliding window with each consecutive 60-mer probe. The expressional confidence of each sliding window (i.e. *p-*values for significant mean differences) was calculated for each sliding window using Student's *t*-test (two-tailed). Custom perl scripts were written to perform these statistical analyses, and also to visualize the expression patterns in bar graphs.

### Database construction

The OsMES database was constructed on a Linux server (FedraCore operating system) to provide information of transcribed regions and gene annotations in the rice mitochondrial genome. In OsMES, information on the transcribed regions and the gene annotations are presented using the genome browser GBrowse version 1.7 [21]. For better accessibility and simplicity, 8245 non-overlapping probes were selected for display. The Generic Feature Format (GFF) file containing the data of predicted transcribed regions by tiling array, six-frame translation and known genic regions in the mitochondrial genome was created using custom perl scripts. The contents of GFF and rice mitochondria genome sequence files were imported into tables via the MySQL database.

### Sequence analysis

Six-frame translation was performed using the getorf program implemented in the EMBOSS v6.0.0 package [22]. Orthologs in 13 plant species, *Arabidopsis thaliana *(NC_001284), *Brassica napus *(NC_008285), *Carica papaya *(NC_012116), *Cucurbita pepo *(NC_014050), *Citrullus lanatus *(NC_014043), *Nicotiana tabacum *(NC_006581), *Beta vulgaris *(NC_002511), *Vitis vinifera *(NC_012119), *Zea mays *(NC_007982), *Tripsacum dactyloides *(NC_008362), *Sorghum bicolor *(NC_008360), *Triticum aestivum *(NC_007579), *Cycas taitungensis *(NC_010303) and *Physcomitrella patens *(NC_007945) were identified by BLASTp from a six-frame translation BLAST library created for each species. ORFs had to be ≥ 70 amino-acids in size in these libraries, as the shortest mitochondrial protein known in rice is orf79 with 79 amino acids [23].

### Northern blot analysis

Mitochondria and mitochondrial RNA were isolated from four-week-old etiolated seedlings and calli (Nipponbare) as described by Tanaka et al. [19] and Zeltz et al. [24], respectively. Of mitochondrial RNA, 3 μg was subjected to northern blot analysis as previously described [25,26]. Digoxigenin (Roche)-labeled probes were obtained by PCR using primers *nad4*, 5'-CCAATATGAGTTTACCCGGC-3' and 5'-GCCATGTTGCACTAAGTTAC-3'; *orf181*, 5'-ACCAGACTACATGCCAAGAC-3' and 5'-GCTAAAATAGATGCCAACCGCA-3'; and *orf490*; 5'-AGATGATCGCAAGTCCACTG-3' and 5'-TTAACCTCCACAATGGAGGC-3'. Signal detection was carried out using LAS-4000 Mini (Fuji Film, Tokyo).

### RT-PCR analysis

Primers used for RT-PCR analysis were listed in Additional file 6.

## List of abbreviations

CMS: cytoplasmic male sterile; GFF: Generic Feature Format; iORFs: inter-genic ORFs; LTR: long terminal repeat; ncRNA: non-coding RNA; ORFs: open reading frames; OsMES: *Oryza sativa *Mitochondrial rna Expression Server; TEs: transposable elements; TU: transcriptional unit.

## Authors' contributions

SF designed the study, carried out the experiments, performed analysis, and drafted the manuscript. TT participated in the experiments. SK, RS, KY, HT and KY constructed databases. KT conceived and supervised the work and edited the manuscript. All authors read and approved the final manuscript.

## Supplementary Material

Additional file 1 **Summary of mitochondrial genome size of different species**. Values on vertical scale are the number of base pairs in each organelle. For metazoans, the average mitochondrial genome size of 1104 species is presented. Chloroplast genome size of *Cucumis melo *is unknown; however, given the constant chloroplast size in angiosperms it is estimated at around 150-160 kb. Click here for file

Additional file 2 **Northern blot analysis of *orf181 *and *orf490 *in calli (C) and seedlings (S)**. RNA expression was undetectable in *orf490 *or *orf181*, the two genes used as untranscribed background region. Hybridization signal of *nad4 *is shown as the positive control. Click here for file

Additional file 3 **Summary of inter-genic ORF (iORF) expression**. Values are given as the mean of all probes within each iORF region. Click here for file

Additional file 4 **RT-PCR analysis of genes 10 iORFs and eight ncRNAs**. RT-PCR analysis was performed using RNA isolated from calli, etiolated seedlings and green seedlings. *rrn18*, 18 S ribosomal RNA. Click here for file

Additional file 5 **Multiple alignment of deduced amino acid sequences of iORF_487 and iORF_502 with known transposable elements**. Upper, alignment of iORF_487 with retroviral aspartyl protease domain of *Arabidopsis thaliana *Athila transposable element (AC022456_8). Lower, alignment of iORF_502 with part of reverse transcriptase domain of *Phyllostachys edulis *(ADB85398.1). Click here for file

Additional file 6 **Spreadsheet information of primers used for RT-PCR analysis**. TU, name of the transcriptional unit; Start, start position of TU; End, end position of TU; Primer 1 and Primer 2, sequence information of primer pair sequences used for RT-PCR analysis. Click here for file
