# Supplementary figures and images for: Transcriptome map of plant mitochondria reveals islands of unexpected transcribed regions
Source: BMC Genomics. 2011 Jun 1;12:279. doi: 10.1186/1471-2164-12-279 (PMC3121727; doi:10.1186/1471-2164-12-279)

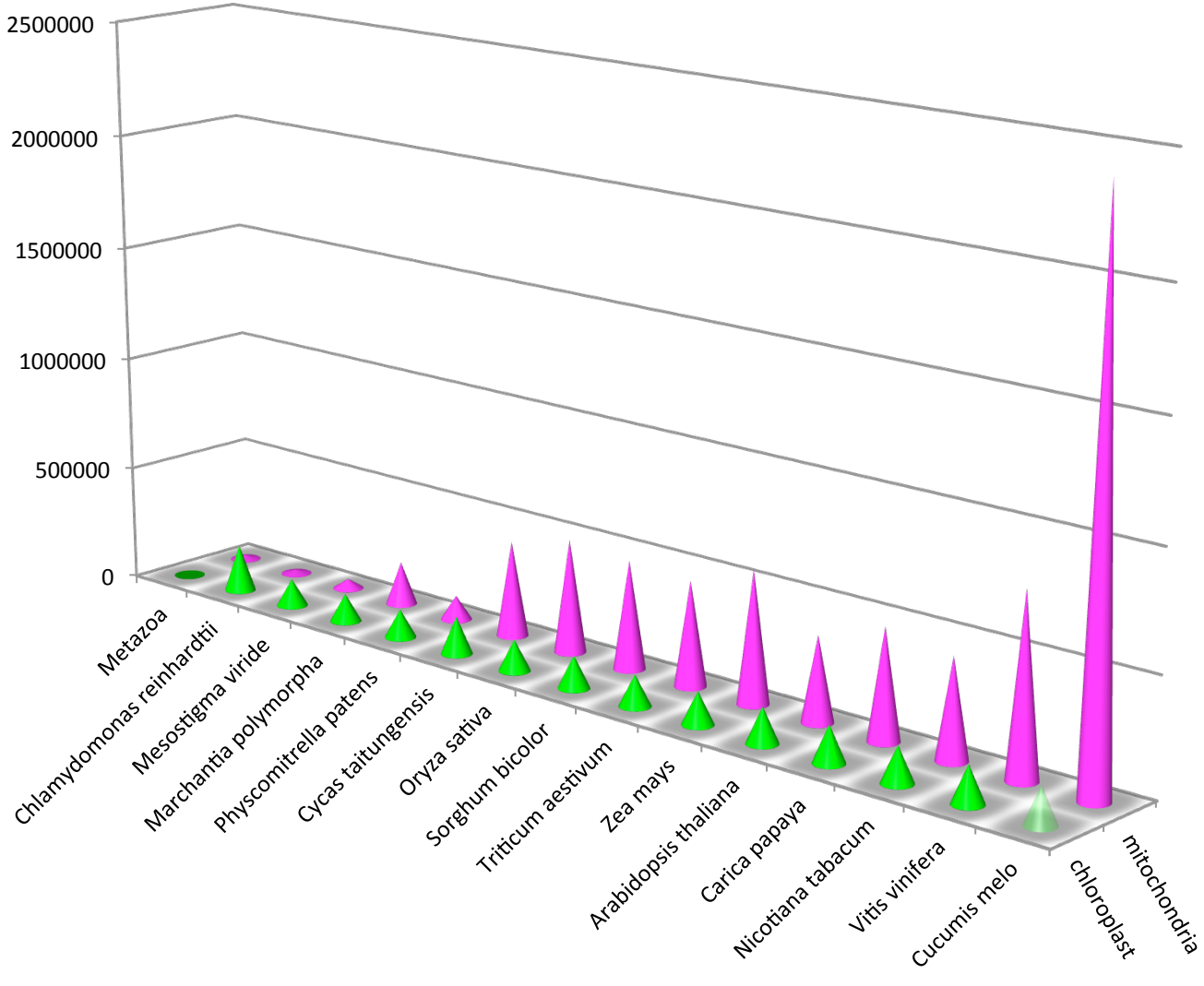

Supplement: Additional file 1 — Summary of mitochondrial genome size of different species. Values on vertical scale are the number of base pairs in each organelle. For metazoans, the average mitochondrial genome size of 1104 species is presented. Chloroplast genome size of Cucumis melo is unknown; however, given the constant chloroplast size in angiosperms it is estimated at around 150-160 kb. [file 1471-2164-12-279-S1.PDF]

|                    |                      |                      |
|--------------------|----------------------|----------------------|
| <b><i>nad4</i></b> | <b><i>orf181</i></b> | <b><i>orf490</i></b> |
| <hr/>              | <hr/>                | <hr/>                |
| <b>S C</b>         | <b>S C</b>           | <b>S C</b>           |

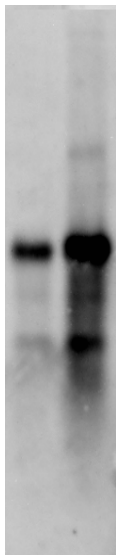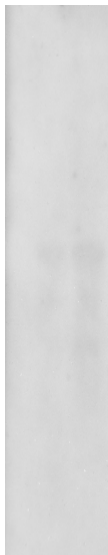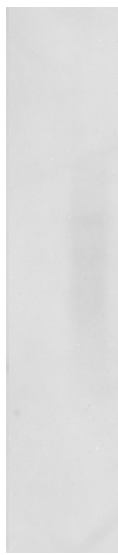

**rRNA**

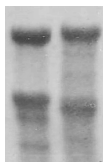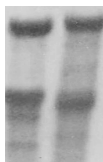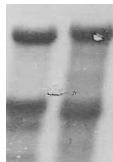

Supplement: Additional file 2 — Northern blot analysis of orf181 and orf490 in calli (C) and seedlings (S). RNA expression was undetectable in orf490 or orf181, the two genes used as untranscribed background region. Hybridization signal of nad4 is shown as the positive control. [file 1471-2164-12-279-S2.PDF]

# Calli

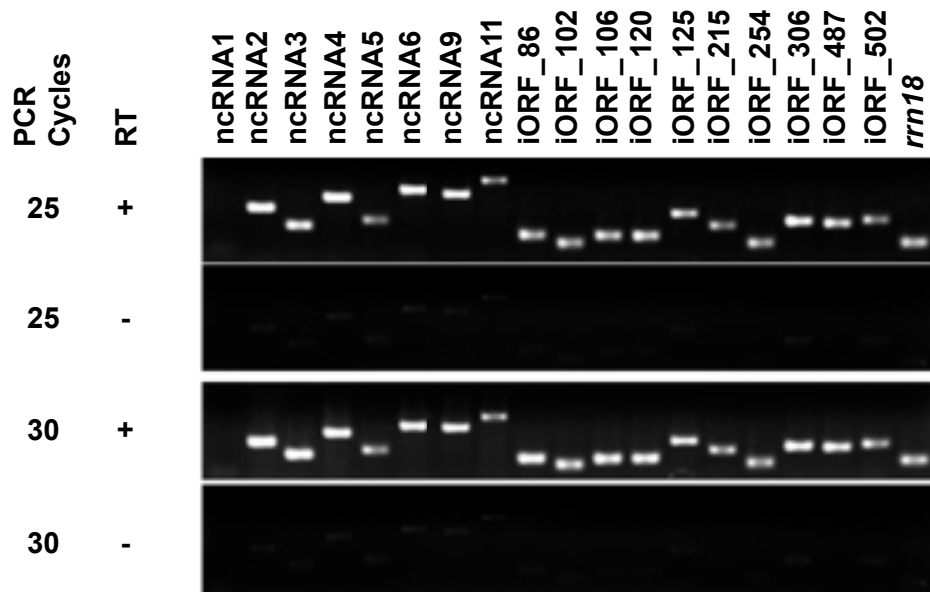

# Etiolated seedlings

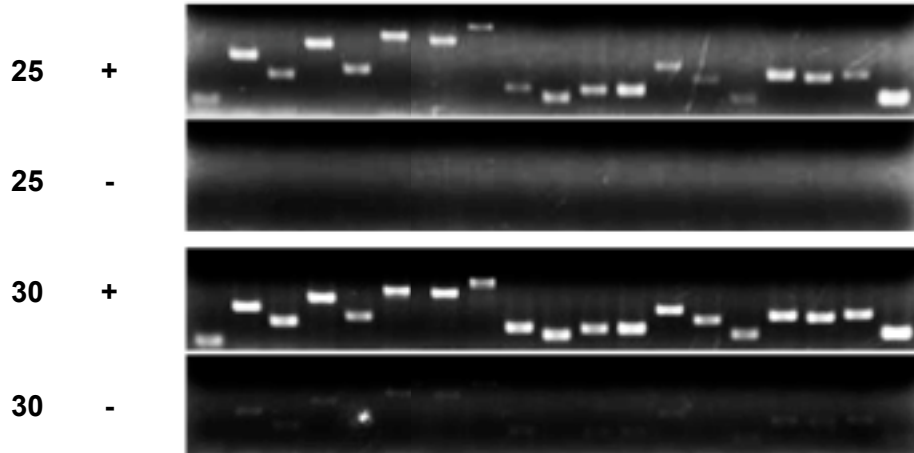

# Green seedlings

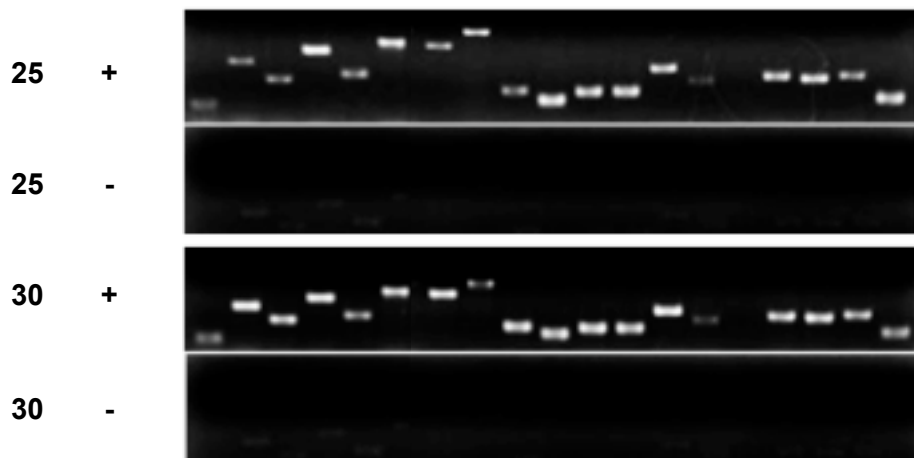

Supplement: Additional file 4 — RT-PCR analysis of genes 10 iORFs and eight ncRNAs. RT-PCR analysis was performed using RNA isolated from calli, etiolated seedlings and green seedlings. rrn18, 18 S ribosomal RNA. [file 1471-2164-12-279-S4.PDF]
