# Supplementary material for: Transcriptome map of plant mitochondria reveals islands of unexpected transcribed regions
Source: BMC Genomics. 2011 Jun 1;12:279. doi: 10.1186/1471-2164-12-279 (PMC3121727; doi:10.1186/1471-2164-12-279)
Supplement: Additional file 5 — Multiple alignment of deduced amino acid sequences of iORF_487 and iORF_502 with known transposable elements. Upper, alignment of iORF_487 with retroviral aspartyl protease domain of Arabidopsis thaliana Athila transposable element (AC022456_8). Lower, alignment of iORF_502 with part of reverse transcriptase domain of Phyllostachys edulis (ADB85398.1). [file 1471-2164-12-279-S5.PDF]

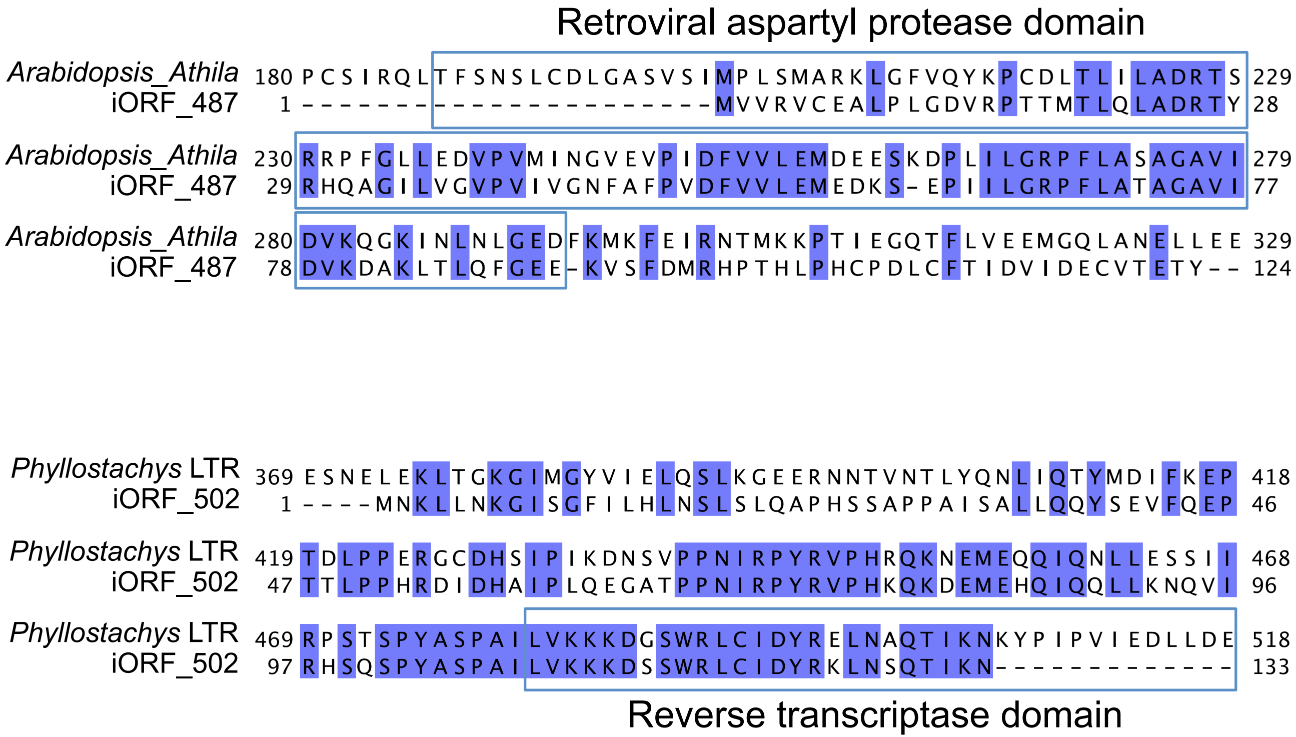
Retroviral aspartyl protease domain
Arabidopsis_Athila 180 PCSIRQLTFSNSLCDLGASVSIMPLSMARKLGFVQYKPCDLTLILADRTS 229
iORF_487 1 ----------------------MVVRVCEALPLGDVRPTTMTLQLADRTY 28
Arabidopsis_Athila 230 RRPFGLLEDVPVMINGVEVPIDFVVLEMDEESKDPLILGRPFLASAGAVI 279
iORF_487 29 RHQAGILVGVPVIVGNFAFPVDFVVLEMEDKS-EPIILGRPFLATAGAVI 77
Arabidopsis_Athila 280 DVKQGKINLNLGEDFKMKFEIRNTMKKPTIEGQTFLVEEMGQLANELLEE 329
iORF_487 78 DVKDAKLTLQFGEE-KVSFDMRHPTHLPHCPDLCFTIDVIDECVTETY-- 124
Phyllostachys LTR 369 ESNELEKLTGKGIMGYVIELQSLKGEERNNTVNTLYQNLIQTYMDIFKEP 418
iORF_502 1 ----MNKLLNKGISGFILHLNSLSLQAPHSSAPPAISALLQQYSEVFQEP 46
Phyllostachys LTR 419 TDLPPERGCDHSIPIKDNSVPPNIRPYRVPHRQKNEMEQQIQNLLESSII 468
iORF_502 47 TTLPPHRDIDHAIPLQEGATPPNIRPYRVPHKQKDEMEHQIQQLLKNQVI 96
Phyllostachys LTR 469 RPSTSPYASPAILVKKKDGSWRLCIDYRELNAQTIKNKYPIPVIEDLLDE 518
iORF_502 97 RHSQSPYASPAILVKKKDSSWRLCIDYRKLNSQTIKN------------- 133
Reverse transcriptase domain
